# Novel QTL and Meta-QTL Mapping for Major Quality Traits in Soybean

**DOI:** 10.3389/fpls.2021.774270

**Published:** 2021-12-08

**Authors:** Heng Chen, Xiangwen Pan, Feifei Wang, Changkai Liu, Xue Wang, Yansheng Li, Qiuying Zhang

**Affiliations:** ^1^Key Laboratory of Soybean Molecular Design and Breeding, Northeast Institute of Geography and Agroecology, Chinese Academy of Sciences, Harbin, China; ^2^Innovation Academy for Seed Design, Chinese Academy of Sciences, Harbin, China; ^3^College of Advanced Agricultural Sciences, University of Chinese Academy of Sciences, Beijing, China

**Keywords:** Soy isoflavone, Soy protein, soybean oil, meta-analysis, whole genome resequencing, quantitative trait locus (loci) (QTL(s))

## Abstract

Isoflavone, protein, and oil are the most important quality traits in soybean. Since these phenotypes are typically quantitative traits, quantitative trait locus (QTL) mapping has been an efficient way to clarify their complex and unclear genetic background. However, the low-density genetic map and the absence of QTL integration limited the accurate and efficient QTL mapping in previous researches. This paper adopted a recombinant inbred lines (RIL) population derived from ‘Zhongdou27’and ‘Hefeng25’ and a high-density linkage map based on whole-genome resequencing to map novel QTL and used meta-analysis methods to integrate the stable and consentaneous QTL. The candidate genes were obtained from gene functional annotation and expression analysis based on the public database. A total of 41 QTL with a high logarithm of odd (LOD) scores were identified through composite interval mapping (CIM), including 38 novel QTL and 2 Stable QTL. A total of 660 candidate genes were predicted according to the results of the gene annotation and public transcriptome data. A total of 212 meta-QTL containing 122 stable and consentaneous QTL were mapped based on 1,034 QTL collected from previous studies. For the first time, 70 meta-QTL associated with isoflavones were mapped in this study. Meanwhile, 69 and 73 meta-QTL, respectively, related to oil and protein were obtained as well. The results promote the understanding of the biosynthesis and regulation of isoflavones, protein, and oil at molecular levels, and facilitate the construction of molecular modular for great quality traits in soybean.

## Introduction

**FIGURE 1:**
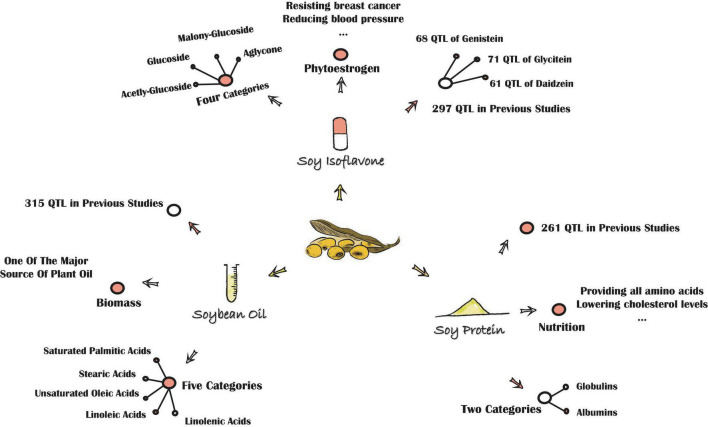
The background of major quality traits in soybean.

Soybean (*Glycine max* L. Merr.), a leguminous plant that originated from China, is one of the most important crops globally, for it is rich in isoflavones, protein, and oil (Figure 1).

Soy isoflavone, a kind of plant secondary metabolite, belongs to a group of 3-phenyl derivatives synthesized by cinnamyl-CoA. Soy isoflavone is generally classified into four main categories: aglycones (AGL), glycosides, acetylglycosides, and malonylglycosides (Sun et al., 2011; Ku et al., 2020). Each category can be further divided into three kinds as well. The AGL include daidzein (DAE), glycitein (GLE), and genistein (GEE). The glucosides (GLU) include daidzin (DA), glycitin (GL), and genistin (GE). The acetylglucosides include acetyldaidzin, acetylglycitin, and acetylgenistin, while the malonylglucosides include malonyldaidzin, malonylglycitin, and malonylgenistin (Sun et al., 2011; Chen et al., 2021). All aglycones are derived from the phenylalanine pathway and can be synthesized into glycosides by the reaction with UDP-Glucose, while glycosides can be synthesized into other two main categories by the increase of the acetyl or malonyl group. Isoflavones are referred to as phytoestrogen (Dixon, 2004), for their structure is similar to estrogen. Nowadays, this secondary metabolite has been widely applied to the clinic, for it could reduce blood pressure, prevent hormone-dependent cancers, alleviate menopausal symptoms, and has numerous other features as well. Isoflavones also play an irreplaceable role in plant disease-resistance, insect resistance, and many other types of stresses (Graham et al., 2007; Yamaguchi et al., 2010), for they are the precursors of major phytoalexins in the system of plant defense responses (Fett, 1984).

Soy protein, generally referred to as the crude protein of soybean, is divided into globulins and albumins based on the solubility patterns. The globulins, categorized as 7S vicilin-type and 11S legumain-type, are the most abundant protein in soybean. Soy protein plays a vital role in human health, for it provides a good balance of essential amino acids for the human diet, and is strongly correlated with lower cholesterol levels and a reduced risk of cardiovascular diseases (Chen et al., 2021). Although breeding soybean cultivars with high protein content has been a major target for decades, the intricate and indistinct genetic background of protein accumulation in plants has hampered this process.

Soybean oil, an important quality trait-like isoflavone and protein, contains five types of fatty acids: saturated palmitic acids, stearic acids, unsaturated oleic acids, linoleic acids, and linolenic acids (Wang et al., 2012). In general, soybean seed is abundant in linoleic acids and linolenic acids, which are essential fatty acids (EFA) for humans. The low saturated fatty acid levels in soybean oil may reduce the risk of coronary diseases and cancer if the ratio of soybean oil in the human diet is increased (Hu et al., 1997). Thus, similar to isoflavone and soy protein, soybean oil could also play a key role in human health.

Due to the crucial importance of these quality traits in plant growth and human health, many studies investigated the accumulation of isoflavones, protein, or oil in soybean seed with final aims to account for the genetic background of these traits, as well as to breed cultivars with high isoflavone, protein, or oil contents (Li et al., 2014). Previous researches suggest that all these quality traits are typically quantitative characters and depend on both environmental and genetic factors (Mao et al., 2013; Zhang et al., 2014; Pei et al., 2018). The discrepancies of these traits between RILs with high, intermediate, and low contents have been presented to be relatively consistent in different environment conditions (Zhang et al., 2014; Huang et al., 2020). Up to now, as the Soybase database (Grant et al., 2010)^1^ shows that there are 261 quantitative trait locus (QTL) associated with protein content and 315 QTL associated with oil content in soybean. In addition, among these 576 QTL, 5 QTL are associated with seed oil plus protein (Chen et al., 2007). Two-hundred ninety-seven QTL related to isoflavones have been detected in soybean including 61 QTL related to seed daidzein, 68 QTL related to seed genistein, 71 QTL related to seed glycitein, and 11 QTL related to seed total isoflavone.

Identifying the number of stable QTL for these quality traits in soybean is essential to understand the genetic factors, unfortunately, there were two inadequacies in previous researches: the low-density genetic map and lack of QTL integration.

Most of the QTL related to soybean isoflavone, protein, or oil contents in previous studies were based on the low-density genetic maps with low throughput molecular markers, such as restriction fragment length polymorphism (RFLP), the variable number of tandem repeats (VNTR), and simple sequence repeat (SSR), which resulted in the low efficiency and accuracy of QTL mapping. Nowadays, with the development of sciences (Jiang, 2000) and DNA sequencing technologies, the QTL mapping can be accomplished based on the high throughput molecular markers and high-density genetic maps. High-throughput sequencing, a next-generation sequencing (NGS) technology, is a powerful technique to construct a high-density genetic map based on a large-scale identification of single nucleotide polymorphisms (SNP) markers (Depristo et al., 2011), and is an efficient tool to map QTL.

Since the QTL is mostly derived from diverse populations and environments in different studies, the integration of QTL is an efficient way to obtain the stable QTL through comparing and combining the QTL from varied researches, as well as of crucial importance in understanding the complicated quantitative characters. The meta-analysis, a method to integrate data from different sources into a single study, has been used mainly by researchers in medical, social, and behavioral sciences (Borenstein et al., 2009). Goffinet and Gerber (2000) reported a technique to combine QTL mapped from multiple independent experiments, and provided a modified Akaike criterion that could be applied to determine which QTL was actually represented by the QTL acquired from different studies. Meta-QTL, the QTL integrated from multiple experiments using meta-analysis and consisting of shorter confidence intervals relative to the original QTL, could be more representative and accurate.

The aims of this research were: (1) to detect more stable QTL associated with isoflavone contents (both individual and total isoflavone), protein, and oil based on a high-density genetic map; (2) to integrate the QTL obtained from the previous researches according to meta-analysis method; (3) to predict candidate genes which may influence the accumulation of these quality traits using gene functional annotation. The results could facilitate elucidating the molecular mechanism of isoflavone, protein, and oil biosynthesis and regulation, as well as constructing molecular modular of great quality traits in soybean.

## Materials and Methods

### Materials and Field Experiment

To construct a recombinant inbred lines (RIL) population for QTL mapping, the single-seed descendent method (Fehr, 1991) was adopted. An F7 population with 160 lines derived from ‘Zhongdou27’ and ‘Hefeng25’ were used, which was named as the ZH RIL population. ‘Hefeng25,’ a higher-yielding cultivar with an isoflavone content of 3199 μg/g, protein content of 40%, and oil content of 20.93%, was mainly planted in Northeast China and planted in Yunnan Province, Hebei Province, and Xinjiang Autonomous Region as well. ‘Zhongdou27’ a late-maturing cultivar with an isoflavone content of 5290 μg/g, protein content of 38.94%, and oil content of 19.94%, is mainly planted in the Huang-Huai-Hai region.

**FIGURE 2:**
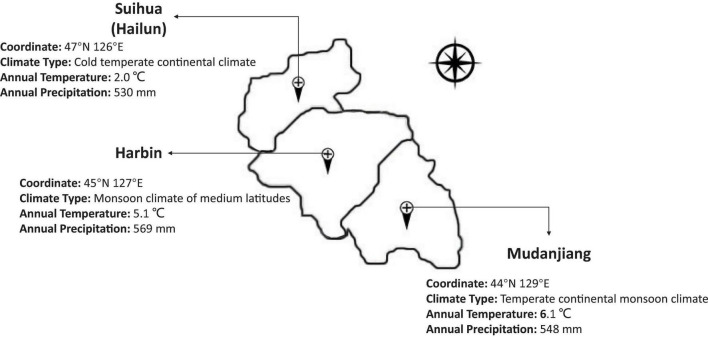
The climate conditions of three experimental stations.

To map the stable QTL related to isoflavones, protein, and oil contents, the 160 RIL and parental lines were planted in three locations with different climates, including Harbin, Mudanjiang, and Hailun in 2020 (Figure 2). A randomized complete block design with three replications was implemented in each location. These materials were sown in rows 3 m long,.65 m wide, and with a distance of.08 m between the individual plants. Field management followed normal soybean production practices for each environmental condition.

All lines of the RIL populations were fully matured in the Harbin and Mudanjiang sites, and only a few lines were not fully matured in the Hailun experimental site, but they were all physiologically matured.

### Phenotype Identification

For the measurement of protein and oil contents in soybean seed, an Infratec 1241 Analyzer (FOSS, Sweden) was used. This is a whole grain analyzer using near-infrared transmittance technology and whose major advantages are rapid, accurate, and non-destructive (Nicolai et al., 2007; Lee et al., 2010). Detailed operations can be acquired on the operating manual of Infratec 1241 Analyzer. Briefly, 80 (or more) soybean seeds were used in the measurement (repeated 10 times), and the result was the average of 10 independent experiments.

For the measurement of isoflavone contents in soybean seed, High-Performance Liquid Chromatography (HPLC) was performed.

The first step was isoflavones extraction. Around.500 g soybean powder was ground by ball milling and sifted by sifter with.002 m aperture, and then placed into a 15 ml centrifuge tube (BIOSHARP, China). The sample was extracted by ultrasonics at 60°C for 30 min, using 9.0 ml 90% (v/v) methanol solution (FUYU, Tianjin, China) as a solvate. The resulting slurry was centrifuged at 5,000 rpm for 5 min, and the supernatant was collected by a 25 ml volumetric flask (TIANBO, Tianjin, China). Then, the sediment was extracted twice, using 6.0 ml 90% (v/v) methanol solution as a solvate. After centrifugation, all the supernatant was collected into the volumetric flask, diluted with 10% (v/v) methanol solution to volume, and mixed intensively. The extracted solution was filtered through a.45 um nylon syringe filter (BIOSHARP, China), and injected into an autosampler vial (SHIMADZU, Japan), and stored at 4°C prior to the follow-up step.

The second step was the HPLC analysis. An LC–10AT HPLC (SHIMADZU, Japan) was used, which equipped a C18 column (4.6 mm × 250 mm, 5 um; AGELA TECHNOLOGIES, Tianjin, China), and controlled by a CLASS-VP V6.1 program. The gradient solution program of HPLC is presented in Table 1, and the conditions are as follows: the solvent flow rate of 1.0 ml/min, the temperature of the column at 40°C, and the SPD-10A detector monitored eluants at 260 nm. The standards of 6 isoflavone components, i.e., DAE, GLE, GEE, DA, GL, and GE (ChromaDex, United States) were used for calculation and analysis. All these six isoflavones were accurately separated in their retention times. The individual isoflavone content was estimated by the specific value of peak area between the standards and samples. The glucoside content was calculated by summing up the contents of DA, GE, and GL, while the aglycone contents were counted by DA, GE, and GL. The sum of the six components, DAE, GLE, GEE, DA, GL, and GE was calculated in the current study, for the glucosides and aglycones are the most major compounds of isoflavones in soybean seed, which could represent the total isoflavone content though it might underestimate it a little bit.

**TABLE 1:** The gradient solution program of high-performance liquid chromatography (HPLC).

Time (min)	Solvent A^a^ (0.1% acetic acid solution)	Solvent B^b^ (0.1% acetic acid acetonitrile solution)
0	90%	10%
12.5	70%	30%
17.5	60%	40%
25	0	100%
28	0	100%
28.1	90%	10%
40	90%	10%

*^a^Solvent A was prepared by ultrapure water.*

*^b^Solvent B was prepared by chromatography pure acetonitrile (KERMEL, Tianjin, China).*

### Phenotype Statistical Analysis

For analyzing the phenotype data of ZH RIL, the *ANOVA*, analysis of heritability (*h*^2^) and coefficient of variance (*CV*) were utilized.

For analyzing the *ANOVA* and *CV* of the isoflavones, protein, and oil contents in soybean seed, the SPPS Statistic 24.0 (IBM, NY, United States) was selected. The statistic model and formula used in the analysis are as follows:


(1) $$ANOVA:y_{ijk}=\mu +G_{i}+L_{j}+{\left(GE\right)}_{ij}+R_{jk}+e_{ijk}$$

where *y*_*ijk*_ represents the contents of these quality traits of *i* the genotype which is planted in *j* the environment under *k* the repeat, μ represents the mean value, *G_i_* represents the effect of *i* the genotype, *L_j_* represents the effect of *j* the environment, *GE*_*ij*_ represents the interaction effect between *i* the genotype and *j* the environment, *R_jk_* represents the interaction effect between *j* the environment and *k* the repeat (block), and *e*_*ijk*_ represents residual error;


(2) $$CV:CV=\frac{\sigma }{m}$$

where σ presents the standard deviation of the quality traits, and *m* presents the mean value.

For the analysis of heritability in the broad sense ($h_{B}^{2}$) of isoflavones, protein, and oil contents in soybean seed, the R package‘lme4’ (Bates et al., 2014) was utilized, which is a program used to determine the maximum likelihood or restricted maximum likelihood (REML) estimates of the parameters in linear mixed-effects models. The formula used to calculate the $h_{B}^{2}$ of each trait is as follows (Wyman and Baker, 1991):


(3) $$h^{2}:h_{B}^{2}=\frac{V_{G}}{V_{G}+V_{E}}=\frac{\sigma_{G}^{2}}{\sigma_{G}^{2}+\left(\sigma_{GE}^{2}/n+\sigma_{E}^{2}/nr\right)}$$

where *V_G* and $\sigma_{G}^{2}$ refers to genetic variance, *V_E* refers to environmental variance, $\sigma_{GE}^{2}$ refers to the variance of genotype and environment interaction in the three experiment locations, $\sigma_{E}^{2}$ refers to the variance of the three environment locations, *n* refers to the number of experiment locations, and *r* refers to the repetitions in each experiment location.

### High-Density Genetic Map Construction

For the high-density genetic map construction, the resequencing with a high coverage level (more than 30-fold) was utilized to sequence the genome of parental lines, and the resequencing (more than 3-fold) was used to sequence the genome of RILs. The conference genome, Wm82.a2.v1, contains 995.708 Mbp of assembled and annotated sequences.

The first step was to construct the DNA libraries of these samples. Total DNA of each parental line and RIL lines was obtained from young and fully developed leaf tissues collected 20 days after emergence, by the cetyltrimethylammonium bromide (CTAB) DNA extraction method. The DNA libraries of 162 lines were sequenced on an Illumina HiSeq 2500 platform (Illumina, CA, United States) and analyzed by an Illumina Casava 2.17 (Illumina, CA, United States) (Levy and Myers, 2016). Then, pair-end sequences, the raw reads as well, with 150 bp long was acquired. The raw reads were filtrated by the quality control (QC) process, and the clean raw reads were obtained. According to the criterion of filtration, the adapter sequences, reads with low-quality (over 50% base with Phred score less than 10), and reads with more than 10% unidentified nucleotides (N) were deleted. After that, the clean reads of these samples were aligned against the reference genome Wm82.a2.v1 (Schmutz et al., 2010)^2^, by the Burrows-Wheeler Aligner (BWA) (Li and Durbin, 2009), which is a program used in comparing the short reads and reference genome. The alignments were formatted and converted into BAM files using SAMtools software (Li et al., 2009), for SNP calling and genotyping.

Secondly, the Genome Analysis Toolkit (GATK) (Mckenna et al., 2010), Picard and Snpeff (Cingolani et al., 2012) was used to detect and annotate the SNP (Depristo et al., 2011) among parental lines and the reference genome, RIL and reference genome, and 2 parental lines, respectively. Detailed operations can be acquired on operating manual^3, 4^. Briefly, the results aligned by BWA were used to delete replication and block the influence of PCR-duplication by Picard. GATK was used in Insert-Deletion (InDel) realignment, base recalibration, and variant calling (include InDel and SNP). The strict standards used to filtrate the SNP were as follows: the SNP cluster (the distance between 2 SNPs less than 5bp), the distance between SNP and InDel less than 5bp, and the distance of 2 InDels less than 10bp (Mckenna et al., 2010).

Finally, the sliding window method with 15 SNPs per window and 1 SNP per step were used to determine the genotype and exchange sites of each RIL and parental line. The bin markers of RILs and parental lines were determined as well. The high-density genetic linkage map was constructed by the bin markers, using a High map (Liu D. et al., 2014).

### Quantitative Trait Locus Identification, Meta-Quantitative Trait Locus Analysis, and Candidate Genes Prediction

To map the QTL of individual and total isoflavone, protein, and oil contents in soybean seed, the CIM (Zeng, 1994) method and R package ‘RQTL’ (Broman et al., 2003; Arends et al., 2010) were used. The threshold of LODs for declaring effective QTLs was determined using a permutation test (PT) with a significance level of *p* < 0.05 (*n* = 1000). QTL with LODs greater than 3.00 would be accepted only. The naming schemes of QTL were: q – trait name – linkage group ID – region number.

To integrate the QTL of isoflavones, protein, and oil, the meta-analysis methods (Goffinet and Gerber, 2000; Borenstein et al., 2009) and BioMercator V4.2.3 software (Arcade et al., 2004) were used. Based on the Soybase database, the information of QTL collected in this research was: QTL names, linkage groups, genetic and physical positions (in Williams 82.a2.v1), LODs, PVE, parental lines, and mapping population types. The QTL collected in this study were renamed according to the following rule: q – trait name – m – QTL number. Then, the QTL was integrated into the public composite genetic map^5^ using BioMercator V4.2.3. Detailed operations can be acquired on the *User Guide BioMercatorV4*^6^. Then, the meta-QTL with more than 2 cm interval lengths were filtered out. Finally, the meta-QTL of isoflavones, protein, and oil were selected from four mathematic models combined with the minimum Akaike Information Criteria (AIC) (Goffinet and Gerber, 2000), and the naming schemes of meta-QTL were: q – trait name – ME – LG – QTL number.

To predict and annotate the candidate genes, the genome sequences corresponding to the QTL intervals were analyzed based on the Phytozome database^7^. Then, the sequences were used in pathway analysis with KEGG database (Kyoto Encyclopedia of Genes and Genomes^8^), and further annotated *via* Basic Local Alignment Search Tool (BLASTX) (Altschul et al., 1997) with the NR database (non-redundant protein sequences^9^) and the Clusters of Orthologous Groups of proteins (COG) database^10^. In addition, the GO database and the SwissPort database^11^ were used in gene functional annotation.

**FIGURE 3:**
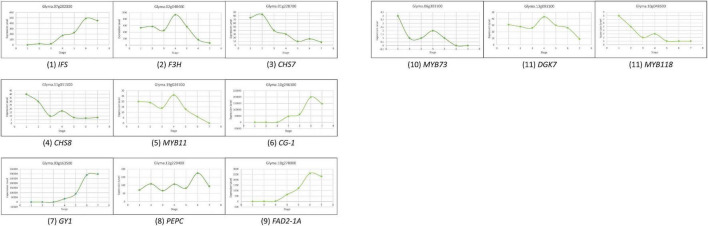
The dynamic variation of reported genes expression in soybean seed during 10 DFA to 42 DAF (on the abscissa, the number 1 refers to 10 DAF, the number 2 refers to 14 DAF, the number 3 refers to 21 DAF, the number 4 refers to 25 DAF, the number 5 refers to 28 DAF, the number 6 refers to 35 DAF, and the number 7 refers to 42 DAF).

To select the candidate genes, based on the RNA-Seq atlas obtained from Soybase (Grant et al., 2010; Severin et al., 2010), we analyzed the dynamic variation of candidate genes expression during the accumulation of isoflavones, protein, and oil. With the combination of gene functional annotation and public transcriptome data, the candidate genes conforming to the following characteristics were supposed to be the major genes: (1) the variation of genes expression level in seed might exhibit the bell-shaped curve, if they could promote the accumulation of these quality traits; (2) the variation of genes expression level in seed might conform to the smiling curve, if they could suppress the accumulation of these quality traits; (3) high level of genes expression during R5 to R8 should be found in the seeds rather than other tissues, if they involve in the biosynthesis of isoflavones and oil directly; (4) high level of genes expression might be found in root nodule, if they could promote the accumulation of protein directly; (5) the variation of genes expression should be ahead of the dynamic change of trait contents significantly, if they are regulated genes or participated in the upstream of the biosynthesis pathways of these traits. Meanwhile, the expression variation of reported genes in soybean seed, *IFS*, *F3H*, *MYB11*, *CHS7*, and *CHS8*, were used as standards to characterize the expression level of different type genes during the accumulation of isoflavones; while the genes *MYB73*, *FAD2-1A*, *DGK7*, and *PEPC*, were selected as background to describe the accumulation of soybean oil. The variation of module formed by *GY1*, *CG-1*, *MYB118*, and *PEPC* represented the dynamic variation of soybean seed oil contents (Table 2 and Figure 3).

**TABLE 2:** The effect of genes selected to characterize the accumulation of isoflavones, protein, and oil in soybean seed.

Trait	Gene Name	Gene ID^a^	Role	Effect^b^	References
Isoflavones	*F3H*	*Glyma.02g048400*	Catalyze the biosynthesis of anthocyanin	−	Chen et al., 2021
	*IFS*	*Glyma.07g202300*	Encode of isoflavone synthase	+	Ku et al., 2020
	*CHS7*	*Glyma.01g228700*	Upstream gene	+	Dhaubhadel et al., 2007
	*CHS8*	*Glyma.11g011500*	Upstream gene	+	Dhaubhadel et al., 2007
	*MYB11*	*Glyma.19g024700*	Regulate element	+	Chen et al., 2021
Soy Protein	*GY1*	*Glyma.03g163500*	Catalyze the biosynthesis of glycinin	+	Nielsen et al., 1989
	*MYB118*	*Glyma.10g048500*	Regulate element	+	Zhang et al., 2009
	*CG-1*	*Glyma.10g246300*	Catalyze the biosynthesis of β-conglycinin	+	Kasuga et al., 1997
	*PEPC*	*Glyma.12g229400*	Encode phosphoenolpyruvate carboxylase	+/−	Hata et al., 1998
Soybean Oil	*MYB73*	*Glyma.06g303100*	Regulate element	+	Liu Y. F. et al., 2014
	*FAD2-1A*	*Glyma.10g278000*	Encode δ-12 fatty acid desaturase 2	+	Bolon et al., 2011
	*DGK7*	*Glyma.13g093100*	Encode diacylglycerol kinase 7	+	Arisz et al., 2009
	*PEPC*	*Glyma.12g229400*	Encode phosphoenolpyruvate carboxylase	+/−	Hata et al., 1998
Protein Plus Oil	*PEPC*	*Glyma.12g229400*	Encode phosphoenolpyruvate carboxylase	+/−	Hata et al., 1998
	*GY1*	*Glyma.03g163500*	Catalyze the biosynthesis of glycinin	+	Nielsen et al., 1989
	*CG-1*	*Glyma.10g246300*	Catalyze the biosynthesis of β-conglycinin	+	Kasuga et al., 1997
	*FAD2-1A*	*Glyma.10g278000*	Encode δ-12 fatty acid desaturase 2	+	Bolon et al., 2011

*^a^Gene ID was based on Wm82.a2.v1 assemblies.*

*^b^Effect: the symbol, “+,” refers to promote; the symbol, “−,” refers to restrain; the symbol, “+/−,” refers to regulate.*

## Results

### Phenotype Identification and Analysis

**FIGURE 4:**
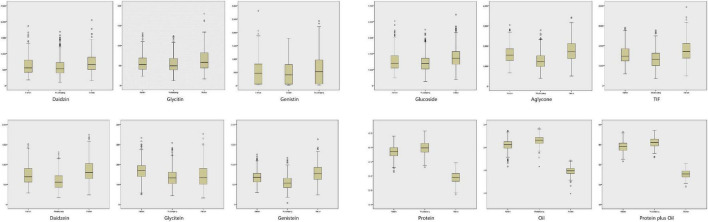
The box plots of 12 quality traits contents in the three experimental locations (Harbin, Mudanjiang, Hailun).

The contents of isoflavone (both individual and total isoflavone), protein and oil of soybean seed in ZH RIL population were determined. As shown in Supplementary Table 1, Figure 4, and Supplementary Figure 1, the contents of these quality traits in all tested environments were: DA (100.4–1864 μg/g), GL (12.11–179.7 μg/g), GE (132.0–2426 μg/g), GLU (258.7–4444 μg/g), DAE (170.3–1743 μg/g), GLE (19.76–353.8 μg/g), GEE (201.6–1630 μg/g), AGL (389.6–3418 μg/g), Total isoflavone (TIF) (699.2–6853 μg/g), protein (31.60–42.90%), oil (17.8–21.7%), protein plus oil (51.5–63.5%).

Supplementary Table 1 and Figure 4 also showed that the variation between the contents of isoflavones in different locations was extremely significant. This consistency and variation appeared in oil and protein as well. The TIF (average value) in the three locations was: 3525 μg/g in Hailun, 3217 μg/g in Harbin, and 2684 μg/g in Mudanjiang. With the decrease of annual average temperature, the TIF was obviously on the rise. This tendency was also significant in the glucoside, aglycone, and 6 isoflavone components. However, this trend was totally opposite in the oil and protein contents, for both the protein and oil contents in Mudanjiang (protein: 39.92%; Oil: 20.59%) were higher, compared with Harbin (protein: 39.28%; Oil: 20.24%) and Hailun (protein: 37.08%; Oil: 19.94%).

According to Supplementary Figure 1, the contents of these quality traits were significantly segregated and showed a typical skewed normal distribution (Skewness≠0), which conformed to the features of quantitative character. The determined lines with a higher or lower transgressive inheritance could be used to further investigate the major genes related to these quality traits, as well as breed cultivars with great quality traits.

Table 3 provides an overview of the statistical analysis. The range of *CV* was 0.03–0.47, indicating that the variation of these phenotypes was significant and reasonable in this study, and this population was suitable for QTL mapping. The great fitting degrees (*F* value: 27.57–454.9) and extremely high significance (*P* < 0.001) of *ANOVA* showed that the environmental effect was extremely significant in these phenotypes; and the$h_{B}^{2}$(0.57–0.89) indicated that the genetic effect was also high-impact.

**TABLE 3:** The statistical analysis of 12 quality traits in the three experimental locations (Harbin, Mudanjiang, Hailun).

Trait	$h_{b}^{2}$ ^a^	*CV* ^b^	*ANOVA^c^*	
			*F* Value	*P* Value
Daidzin	0.88	0.47	29.62	<0.001
Glycitin	0.89	0.41	16.09	<0.001
Genistin	0.77	0.40	39.45	<0.001
Glucoside	0.82	0.40	38.46	<0.001
Daidzein	0.89	0.37	125.61	<0.001
Glycitein	0.63	0.47	27.57	<0.001
Genistein	0.82	0.31	160.33	<0.001
Aglycone	0.85	0.32	128.09	<0.001
TIF	0.87	0.33	78.97	<0.001
Protein	0.57	0.05	365.61	<0.001
Oil	0.82	0.03	170.51	<0.001
Protein+Oil	0.65	0.04	454.85	<0.001

*^a^$h_{b}^{2}$ means heritability in the broad sense.*

*^b^CV means the coefficient of variance.*

*^c^ANOVA means the analysis of variance; P value < 0.001 means an extremely high significance.*

### High-Density Genetic Map Construction

**FIGURE 5:**
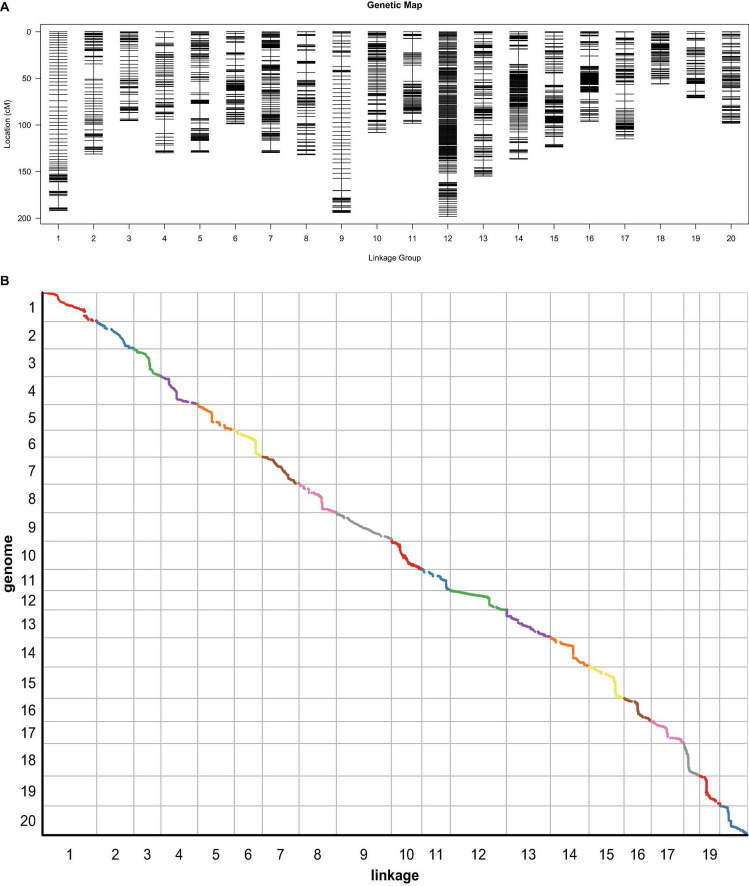
The genetic map based on the ZH recombinant inbred lines (RIL) population. **(A)** The high-density genetic map. **(B)** The collinearity analysis between genetic map and genome. The slope of the line in each block means the Spearman correlation coefficient.

Based on the resequencing technology, a total of 561.95 Gbp clean data, with a Q30 ratio of more than 90%, of the ZH RIL population were collected, including Zhongdou27 (37.43 Gbp), Hefeng25 (27.29 Gbp), and 160 RILs (501.23 Gbp). Furthermore, a total of 1,283,813 SNP between the parental lines were detected, which included 1,093,273 SNP also detected in RIL. There were 283,855 InDel among the parental lines and 101,602.8 InDel per line between the RIL and parental lines. Finally, a total of 5,338 bin markers were obtained based on the sliding window method with these data, and a total length of 2,487.17 cM high-density genetic map with an average distance of.47 cm was constructed (Figure 5A and Table 4). The collinearity analysis between the linkage map and reference genome showed that the Spearman correlation coefficients were almost greater than 0.99, which revealed that the linkage map was highly accurate (Figure 5B).

**TABLE 4:** The basic information of the high-density genetic map.

LG^a^	Total Bin Marker	Total^b^ Distance (cM)	Average Distance (cM)	Max Gap (cM)	Gaps < 5cM (%)	Spearman^c^
LG01	165	191.75	1.17	12.98	95.73%	0.9968
LG02	164	131.08	0.8	16.32	94.48%	0.9986
LG03	183	95.42	0.52	6.88	98.35%	0.986
LG04	167	129.89	0.78	15.41	95.18%	0.9858
LG05	175	129.11	0.74	16	91.38%	0.9991
LG06	304	98.83	0.33	10.94	98.68%	0.9982
LG07	189	129.66	0.69	10.94	98.94%	0.9997
LG08	242	131.87	0.55	11.44	96.68%	0.9992
LG09	119	193.92	1.64	12.98	88.14%	0.9993
LG10	185	107.97	0.59	6.06	97.83%	0.996
LG11	138	98.17	0.72	17.23	97.81%	0.9993
LG12	268	198.28	0.74	10.04	99.63%	0.9998
LG13	373	154.91	0.42	10.52	74.73%	0.8832
LG14	277	136.6	0.49	16.4	97.83%	0.9995
LG15	436	123.64	0.28	12.98	99.31%	0.9864
LG16	747	96.11	0.13	11.01	98.53%	0.9802
LG17	150	114.96	0.77	10.22	97.32%	0.9983
LG18	443	56.05	0.13	5.27	99.32%	0.9879
LG19	385	70.77	0.18	8.16	99.48%	0.8647
LG20	228	98.2	0.43	6.06	98.68%	0.9915
Total	5338	2487.17	0.47	17.23	74.73%	

*^a^LG means the linkage group ID.*

*^b^Total distance means the total genetic distance of a linkage group.*

*^c^Spearman means the rank correlation coefficient.*

### Quantitative Trait Loci Identification

A total of 41 QTL located on 12 linkage groups (LG) were discovered through CIM in this study, including 38 novel QTL and three reported QTL. There were 27 QTL related to isoflavones (six isoflavone components, glucoside, aglycone, and TIF), seven QTL associated with protein, four QTL linked with oil, and three QTL involved in protein plus oil (Table 5). Two novel QTL, qDAE0403 and qGL1102, were stable, for they exhibited high LODs in all environmental conditions. Nine novel QTL, including qDA0403, qGLU0403, qAGL0403, qTIF0403, qAGL0501, qDA0502, qDAE0502, qGLU0502, and qTIF0502, were sub-stable QTL for they had high LODs in two environmental conditions.

**TABLE 5:** QTL mapping of 12 quality traits for the ZH RIL populations in the three experimental locations.

Location	Trait	QTL	LG^a^	Genetic (cM)^b^	Physical^c^	LOD^d^	ADD^e^	PVE%^f^
Harbin	Daidzin	qDA0403	4	129.76–129.89	50734438–51188701	17.69	–114.51	4.34
		qDA0502	5	128.53–129.11	41630011–42232887	15.01	–139.85	4.81
	Genistin	qGE0502	5	128.53–129.11	41630011–42232887	7.20	–115.86	2.89
	Glycitin	qGL1102	11	32.54–32.54	7769308–8050484	18.89	–11.13	5.13
	Daidzein	qDAE0403	4	129.76–129.89	50734438–51188701	13.24	–299.01	3.51
		qDAE0502	5	128.53–129.11	41630011–42232887	18.07	–115.36	4.40
	Genistein	qGEE0402	4	127.66–128.04	50393326–50647825	9.99	–6.03	2.83
		qGEE0501	5	128.41–128.53	41610737–41670097	9.57	6.00	3.25
	Glycitein	qGLE0402	4	127.66–128.04	50393326–50647825	10.41	–181.55	2.84
		qGLE0501	5	128.41–128.53	41610737–41670097	6.03	–5.11	2.03
	Glucoside	qGLU0403	4	129.76–129.89	50734438–51188701	12.50	–118.74	3.47
		qGLU0502	5	128.53–129.11	41630011–42232887	10.05	–250.43	3.70
	Aglycone	qAGL0403	4	129.76–129.89	50734438–51188701	8.66	–213.96	2.70
		qAGL0501	5	128.41–128.53	41610737–41670097	7.34	0.14	1.37
	TIF	qTIF0403	4	129.76–129.89	50734438–51188701	13.60	–512.97	3.59
		qTIF0502	5	128.53–129.11	41630011–42232887	15.51	–543.09	4.02
	Protein	qPRO0201	2	14.68–27.00	7032252–12057589	4.10	0.18	0.41
		qPRO0902	9	12.21–12.53	4821122–4855330	3.29	0.22	0.63
		qPRO1302	13	123.16–123.48	39305991–39651247	3.48	–0.29	1.07
		qPRO1701	17	7.91–7.91	4754502–7280175	3.20	–0.26	0.86
		qPRO2001	20	59.79–60.11	35720194–36775443	3.93	–0.29	1.04
	Oil	qOIL0501	5	128.41–128.53	41610737–41670097	7.41	–165.46	2.36
		qOIL1901	19	70.45–70.77	45686265–49331240	7.21	0.17	2.05
	Protein+Oil	qPO0901	9	0.00–0.00	550666–1162614	4.51	0.33	1.05
Mudanjiang	Daidzin	qDA0502	5	128.53–129.11	41630011–42232887	13.35	–144.11	4.84
	Genistin	qGE0501	5	128.41–128.53	41610737–41670097	6.25	–114.31	2.52
		qGE1001	10	1.62–2.25	660536–3172923	6.91	–119.10	2.74
	Glycitin	qGL1102	11	32.54–32.54	7769308–8050484	16.20	–11.44	5.28
	Daidzein	qDAE0403	4	129.76–129.89	50734438–51188701	8.15	–83.95	2.66
		qDAE0502	5	128.53–129.11	41630011–42232887	12.29	–107.53	4.37
	Genistein	qGEE1801	18	46.91–46.91	52688683–52735759	5.75	–56.14	1.70
	Glycitein	qGLE0903	9	178.90–179.21	40194590–40275084	3.03	–17.41	0.88
	Glucoside	qGLU0502	5	128.53–129.11	41630011–42232887	9.05	–178.69	3.01
	Aglycone	qAGL0502	5	128.53–129.11	41630011–42232887	10.49	–261.93	3.83
	TIF	qTIF0502	5	128.53–129.11	41630011–42232887	10.04	–434.81	3.60
	Protein	qPRO1301	13	116.78–117.10	38932430–39299927	2.75	–0.33	1.16
	Oil	qOIL1201	12	146.30–147.27	33222958–33941964	4.21	0.09	0.71
	Protein+Oil	qPO1301	13	116.78–117.10	38932430–39299927	4.31	–0.33	1.30
Hailun	Daidzin	qDA0403	4	129.76–129.89	50734438–51188701	10.26	–115.06	3.70
	Genistin	qGE0401	4	119.92–119.92	49356058–49550267	8.61	–111.13	2.35
	Glycitin	qGL1102	11	32.54–32.54	7769308–8050484	11.00	–14.34	5.07
	Daidzein	qDAE0403	4	129.76–129.89	50734438–51188701	9.02	–206.66	3.47
		qDAE0501	5	128.41–128.53	41610737–41670097	5.73	–183.95	2.75
	Genistein	qGEE0401	4	119.92–119.92	49356058–49550267	6.98	–69.51	2.42
		qGEE0502	5	128.53–129.11	41630011–42232887	4.96	–15.27	1.92
	Glycitein	qGLE0403	4	129.76–129.89	50734438–51188701	7.37	–220.64	2.81
	Glucoside	qGLU0403	4	129.76–129.89	50734438–51188701	9.57	–119.43	3.52
	Aglycone	qAGL0403	4	129.76–129.89	50734438–51188701	5.03	–19.94	3.28
		qAGL0501	5	128.41–128.53	41610737–41670097	7.71	–102.69	2.94
	TIF	qTIF0403	4	129.76–129.89	50734438–51188701	8.76	–427.29	3.38
		qTIF0501	5	128.41–128.53	41610737–41670097	5.74	–386.01	2.76
	Protein	qPRO1101	11	24.40–24.72	4850837–5750235	2.21	0.38	0.83
	Oil	qOIL1702	17	106.90–107.35	38474967–38635444	2.47	–0.21	2.05

*^a^LG means the linkage group ID.*

*^b^Genetic means the Genetic distance between the Bin makers projected in the public map (Wm82.a2.v1).*

*^c^Physical means the Physical distance between the Bin makers based on the reference genome (Wm82.a2.v1).*

*^d^LOD means the logarithm of odd of QTL.*

*^e^ADD means the additive effect of QTL.*

*^f^PVE% means the phenotypic variance explained by QTL.*

Among the QTL associated with isoflavones contents, the max LOD of QTL, the phenotypic variance explained by QTL (PVE), and the additive effects contributed by QTL ranged from 3.03 to 18.89, 0.88 to 5.28%, and –5.11 to –543.09, respectively. The properties of QTL associated with soy protein were: max LOD (2.21 to 7.34), PVE (0.41 to 2.05%), and additive effects (–0.457 to 0.377). As for the QTL associated with soybean oil, the range of max LOD, PVE, and additive effects of QTL were 2.21 to 7.34, 0.71 to 2.05, and –0.46 to 0.38, respectively. The range of LOD, PVE, and additive effects of QTL related to protein plus oil were 2.72 to 4.51, 0.92 to 1.16%, and −0.457 to 0.334, respectively.

**FIGURE 6:**
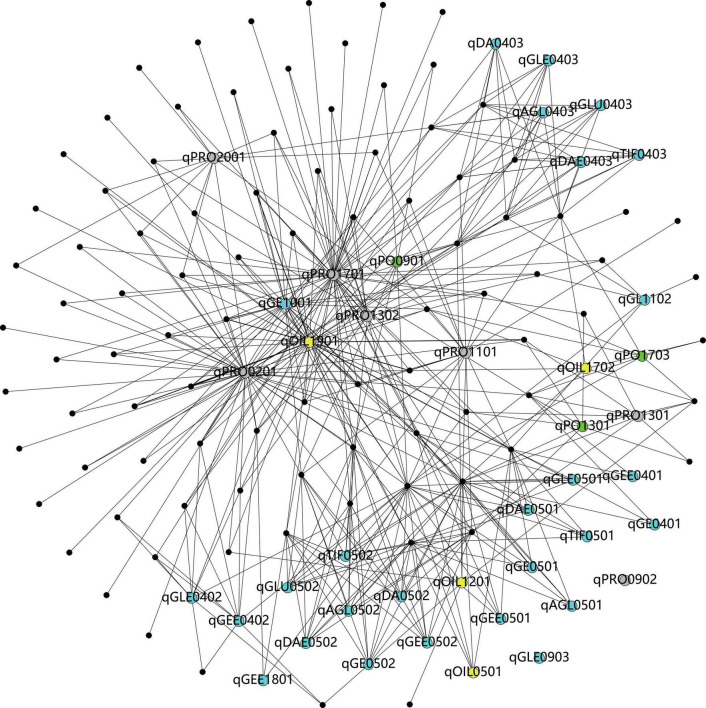
The visualization of correlations between pathways and quantitative trait locus (QTL) (the black dots refer to pathways, the blue dots refer to the QTL associated with isoflavones, the gray dots present the QTL linked with protein, the yellow dots mean the QTL related to oil, and the green dots present to the QTL associated with protein plus oil).

**FIGURE 7:**
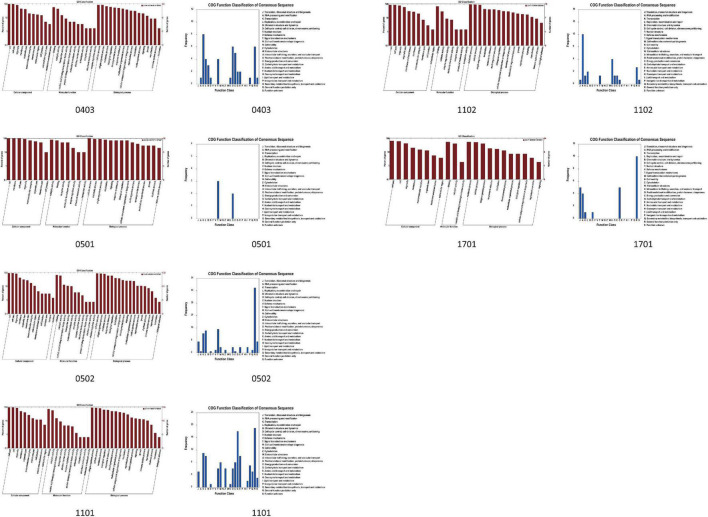
The histogram of function classification of QTL based on the GO and Clusters of Orthologous Groups of proteins (COG) database (the number 0403 refers to the region of qDAE0403, qDA0403, qGLU0403, qAGL0403, and qTIF0403. The number 0501 refers to the region of qAGL0501. The number 0502 refers to the region of qDA0502, qDAE0502, qGLU0502, and qTIF0502. The numbers, 1101, qq02, and 1702, refer to the region of qPRO1101, qGL1102, and qOIL1702, respectively).

On the basis of the GO and Phytozome database, a total of 2,203 genes were obtained in this study (Table 6). According to the pathway analysis with the Kyoto Encyclopedia of Genes and Genomes (KEGG) database, a total of 93 pathways which involved 39 QTL (except for qPRO0902 and qGLE0903) and 830 genes were analyzed, including several pathways directly related to the biosynthesis of isoflavones (e.g., phenylalanine metabolism, flavonoid biosynthesis), oil (e.g., TCA cycle, fatty acid metabolism), and protein (e.g., tyrosine metabolism), as well as some pathways who might regulate the biosynthesis (e.g., aminoacyl-tRNA biosynthesis, basal transcription factors). The results obtained from the correlation analysis (Bastian et al., 2009) between pathways and QTL were summarized in Figure 6. Further combining with the results of annotation based on GO, SwissPort, NR, and COG database, 3,536 protein and 3,093 kinds of function related to these candidate genes were predicted. The function classification of stable and sub-stable QTL is summarized in Figure 7.

**TABLE 6:** The gene functional annotation of QTL.

LG^a^	Region	QTL	Candidate Gene^b^	Selected Gene^c^	COG	GO	KEGG	SwissPort	Nr	Location^d^
2	1	qPRO0201	401	106	233	585	113	549	647	H
4	1	qGE0401	24	8	21	39	7	32	45	HL
		qGEE0401	24	8	21	39	7	32	45	HL
	2	qGEE0402	22	9	29	54	15	49	54	H
		qGLE0402	22	9	29	54	15	49	54	H
	3	qDA0403	54	21	21	60	8	65	80	H, HL
		qDAE0403	54	21	21	60	8	65	80	H, HL, M
		qGLE0403	54	21	21	60	8	65	80	HL
		qGLU0403	54	21	21	60	8	65	80	H, HL
		qAGL0403	54	21	21	60	8	65	80	H, HL
		qTIF0403	54	21	21	60	8	65	80	H, HL
5	1	qGE0501	6	5	5	10	9	10	10	M
		qDAE0501	6	5	5	10	9	10	10	HL
		qGEE0501	6	5	5	10	9	10	10	H
		qGLE0501	6	5	5	10	9	10	10	H
		qAGL0501	6	5	5	10	9	10	10	H, HL
		qTIF0501	6	5	5	10	9	10	10	HL
		qOIL0501	6	4	5	10	9	10	10	H
	2	qDA0502	68	30	89	139	35	123	149	H, M
		qGE0502	68	30	89	139	35	123	149	H
		qDAE0502	68	30	89	139	35	123	149	H, M
		qGEE0502	68	30	89	139	35	123	149	HL
		qGLU0502	68	30	89	139	35	123	149	H, M
		qAGL0502	68	30	89	139	35	123	149	M
		qTIF0502	68	30	89	139	35	123	149	H, M
9	1	qPO0901	76	31	38	99	40	100	111	H
	2	qPRO0902	3	1	0	0	0	1	4	H
	3	qGLE0903	5	2	7	9	0	10	10	M
10	1	qGE1001	298	73	173	392	102	389	448	M
11	1	qPRO1101	127	34	79	158	44	152	169	HL
	2	qGL1102	37	9	30	70	43	70	82	H, HL, M
12	1	qOIL1201	45	12	46	79	17	81	96	M
13	1	qPRO1301	43	25	27	59	13	58	69	M
		qPO1301	43	15	27	59	13	58	69	M
	2	qPRO1302	47	22	23	46	10	48	74	H
17	1	qPRO1701	312	82	221	439	147	419	501	H
	2	qOIL1702	17	4	7	24	11	24	29	HL
	3	qPO1703	39	14	24	54	9	51	67	HL
18	1	qGEE1801	3	2	1	3	3	3	3	M
19	1	qOIL1901	468	132	325	637	178	629	738	H
20	1	qPRO2001	101	30	75	137	26	123	150	H

*^a^LG means the linkage group ID.*

*^b^Candidate Gene means the Gene numbers in the QTL annotated by the GO and Phytozome database.*

*^c^Selected Gene means the Gene numbers in the QTL selected from the candidate genes by the combination of gene functional annotation and public transcriptome data.*

*^d^Location means the experimental location in which the QTL mapped, where H refer to Harbin, and HL refer to Hailun, and M refer to Mudanjiang.*

Further integrating the expression analysis with public transcriptome data, a total of 2,203 candidate genes were selected, including 143 genes related to isoflavones (6 isoflavone components, glucoside, aglycone, and TIF), 300 genes associated with protein, 152 genes involved in oil, and 60 genes linked with protein plus oil (Table 6). The expression dynamic variation of these genes during 10 DAF to 42 DAF were conformed to our hypothesis. The stable QTL, qDAE0403, contain 21 genes; and the other one, qGL1102, contain nine genes (Table 7). The major genes of nine sub-stable QTL and three reported QTL were predicted as well (Table 7).

**TABLE 7:** The selected genes of stable and sub-stable QTL.

LG^a^	Region	Physical^b^	QTL	Location^c^	Selected Gene^d^
4	3	50734438–51188701	qDAE0403	H, HL, M	*Glyma.04G238900*
					*Glyma.04G239300*
					*Glyma.04G239600*
			qDA0403	H, HL	*Glyma.04G240000*
					*Glyma.04G240200*
					*Glyma.04G240400*
					*Glyma.04G240600*
					*Glyma.04G240900*
			qGLU0403	H, HL	*Glyma.04G241000*
					Glyma.04G241100
					Glyma.04G241800
					Glyma.04G241900
			qAGL0403	H, HL	Glyma.04G242300
					Glyma.04G242400
					Glyma.04G242500
					Glyma.04G242700
					Glyma.04G242800
			qTIF0403	H, HL	Glyma.04G243000
					Glyma.04G243600
					Glyma.04G244000
					Glyma.04G244100
5	1	41610737–41670097	qAGL0501	H, HL	*Glyma.05G241100*
					Glyma.05G241200
					Glyma.05G241400
					Glyma.05G241500
					Glyma.05G241600
	2	41630011–42232887	qDA0502	H, M	*Glyma.05G241800*
					Glyma.05G242000
					Glyma.05G242800
					Glyma.05G243200
					Glyma.05G243300
					Glyma.05G243400
					Glyma.05G243600
			qDAE0502	H, M	Glyma.05G243900
					Glyma.05G244300
					Glyma.05G244400
					Glyma.05G244500
					Glyma.05G244600
					Glyma.05G244800
					Glyma.05G245000
					Glyma.05G245100
			qGLU0502	H, M	Glyma.05G245300
					Glyma.05G245800
					Glyma.05G246400
					Glyma.05G246700
					Glyma.05G247000
					Glyma.05G247100
					Glyma.05G247200
			qTIF0502	H, M	Glyma.05G247300
					Glyma.05G247700
					Glyma.05G247800
					Glyma.05G248000
					Glyma.05G248300
					Glyma.05G248500
					Glyma.05G248600
					Glyma.05G248900
9	2	4821122–4855330	qPRO0902	H, RP	*Glyma.09G054500*
11	1	4850837–5750235	qPRO1101	HL, RP	*Glyma.11G068000*
					Glyma.11G067300
					Glyma.11G064300
					Glyma.11G076000
					Glyma.11G067800
					Glyma.11G070200
					Glyma.11G070900
					Glyma.11G075700
					Glyma.11G070800
					Glyma.11G075500
					Glyma.11G075300
					Glyma.11G069400
					Glyma.11G071700
					Glyma.11G071600
					Glyma.11G069600
					Glyma.11G074700
					Glyma.11G073000
					Glyma.11G065200
					Glyma.11G076300
					Glyma.11G071500
					Glyma.11G070000
					Glyma.11G067400
					Glyma.11G066500
					Glyma.11G073500
					Glyma.11G074200
					Glyma.11G076200
					Glyma.11G064900
					Glyma.11G066600
					Glyma.11G072300
					Glyma.11G066200
					Glyma.11G066000
					Glyma.11G066100
					Glyma.11G066400
					Glyma.11G066300
	2	7769308–8050484	qGL1102	H, HL, M	*Glyma.11G103900*
					Glyma.11G105800
					Glyma.11G104100
					Glyma.11G103300
					Glyma.11G102600
					Glyma.11G102700
					Glyma.11G105000
					Glyma.11G105100
					Glyma.11G105500
17	2	38474967–38635444	qOIL1702	HL, RP	*Glyma.17G231300*
					Glyma.17G230100
					Glyma.17G230200
					Glyma.17G229800

*^a^LG means the linkage group ID.*

*^b^Physical means the Physical distance between the Bin makers based on the reference genome (Wm82.a2.v1).*

*^c^Location means the experimental location in which the QTL mapped, where H refer to Harbin, and HL refer to Hailun, and M refer to Mudanjiang, and RP refer to report in previous studies, and MT refer to Meta-QTL.*

*^d^Selected Gene means the Gene numbers in the QTL selected from the candidate genes by the combination of gene functional annotation and public transcriptome data.*

### Meta-Analysis

**FIGURE 8:**
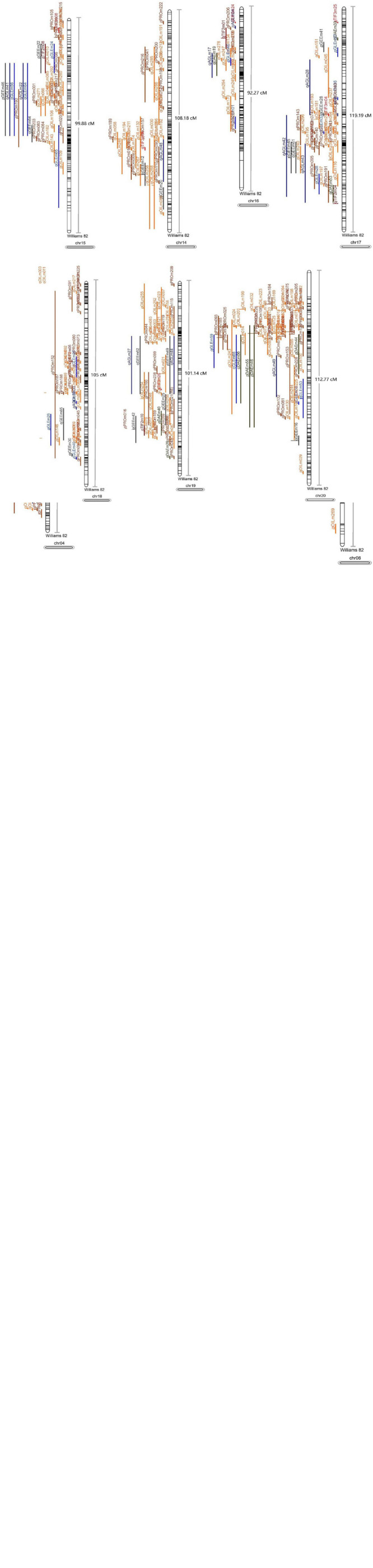
The projection of the QTL collected in this research (the black lines in chromosomes refer to the molecular markers and the other lines refer to the QTL used in meta-analysis).

A total of 1,034 QTL were collected from previous studies and used in meta-analysis (Figure 8 and Supplementary Table 2), including 368 QTL associated with seed oil, 316 QTL related to seed protein, and 350 QTL correlated to isoflavones. We mapped 212 meta-QTL, respectively, related to isoflavones, oil, and protein, which were distributed on all linkage groups (Supplementary Table 3), and this meta-QTL was generated from more than 1 original QTL. Of this meta-QTL, 55 QTL were located on the interval length with no more than 1 cm.

There was 7 meta-QTL associated with aglycone, which were located on the 5 LG (6th, 7th, 10th, 13th, and 17th LG) and whose AIC value varied from 7.29 to 446.06. Of these QTL, qAGLME061 derived from two original QTL had the shortest interval length (0.98 cm). In addition, only 1 meta-QTL (qTIF2ME031) linked with TIF2 and four QTL associated with TIF3 were mapped in this study.

There was five meta-QTL related to DAE with a low AIC value (13.88 to 64.92), which were mapped in the 2 LG including 4th and 5th LG. The interval length of these QTL ranged from 1.26 to 2.00 cM.

A total of 28 meta-QTL of GEE was mapped in this study, which was in 8 LG (4th, 5th, 6th, 7th, 8th, 12th, 13th, and 17th LG). Of these QTL, qGEEME121 was located in a 0.58 cM region and qGEEME124 contained a 0.61 cM confidence interval, which was the shortest interval length. In addition, the AIC of qGEEME043 (11.61) and qGEEME044 (11.61) were the lowest.

This study mapped 26 meta-QTL linked to GLE from 70 original QTL. These QTL are located on 11 LG, including 1st, 3rd, 6th, 7th, 8th, 9th, 10th, 11th, 12th, 17th, and 20th LG, with an AIC less than 422.22. The region of qGLEME011 was only 0.6 cM length.

Based on 309 QTL, 69 meta-QTL related to oil contents were mapped, which were located on the 17 chromosomes (except for the 10th, 11th, and 20th chromosomes). Of these QTL, 24 QTL were located in the regions with no more than 1 cm length. Furthermore, the QTL, qOILME131, and qOILME142 contained only 0.04 cm intervals.

We obtained 73 QTL related to protein-based on meta-analysis, which was located on 19 LG (except for 14th LG). The QTL, qPROME152 contained an 0.16 cm region, and qPROME114 was in a 0.19 cm region. A total of 2 QTL (qPROME162 and qPROME163) exhibited the lowest AIC value (8.13).

## Discussion

### Meta-Analysis

This study showed that the meta-analysis method can map stable QTL, which can offset the limitation of traditional approaches of QTL mapping: most of QTL were environmental-dependent loci which might not be effective in the environmental conditions and difficult to utilize in breeding. In this study, a total of 212 meta-QTL was obtained in this research, including 122 stable QTL. Of them, 70 meta-QTL related to isoflavones were mapped for the first time by the meta-analysis method in investigating the genetic background of isoflavones accumulation. Moreover, 69 QTL associated with oil and 73 QTL linked with protein were also acquired in this study. Several meta-QTL associated with oil or protein were mapped. The present study not only examined more previous research data and used a higher quality genetic map but also obtained more meta-QTL and more accurate intervals, compared with the previous researches. Therefore, based on the meta-analysis to integrate QTL is an efficient way to obtain the stable QTL, as well as of crucial importance in understanding the complicated quantitative characters.

The mean interval length of these meta-QTLs was 1.50 cm while the length of original QTL collected from the previous studies was 11.00 cm. This indicated that the interval length of meta-QTL was obviously shorter compared with the original QTL. Moreover, of these 212 meta-QTL, 122 QTL were stable and consentaneous QTL. The original QTL was derived from two or three environments in general, making it difficult to map the stable QTL. The meta-analysis methods could remedy the limitation of the traditional methods for QTL mapping, for it could integrate the QTL based on different parental lines, diverse mapping populations, multiple environments, and various experimental designs. Thus, the meta-analysis methods and integration of QTL are powerful and assistant tools for the investigation of QTL, which could effectively promote the process of QTL mapping.

There were two stable meta-QTL: qGEEME062 and qAGLME061. The qGEEME062 (112.39–113.38 cm) was derived from qGEEm01, qGEEm37, and qGEEm44 (Primomo et al., 2005; Smallwood et al., 2014; Wang et al., 2015), and involved three pairs of parental lines and 11 kinds of environmental conditions; and the *qAGLME061* (112.4–113.38 cm) was mapped from qAGLm02 and qAGLm18 (Primomo et al., 2005; Smallwood et al., 2014), and was obtained from the 4 parental lines and 8 environments. Since these two QTLs were almost overlapped, this study suggested that they may contain the common major genes. Furthermore, qGEEME062 which was related to the contents of GEE, might influence the aglycone contents, for the contents of genistein makes up a large proportion of the aglycone contents. The gene functional annotation provided further evidence for the assumption that: the Glyma.06G207900, encoding a protein involved in the glucose catabolic process, may be the common major gene of these two QTL.

As same as qGEEME062 and qAGLME061, qAGLME132 (101.14–103.13 cm) and qGEEME133 (101.07–103.07 cm) were supposed to be the same QTL or (and) contained the common major genes in this research as well. The reasons are: (1) they were partly overlapped; (2) the phenotypes of these two QTL were significantly correlated. Furthermore, the gene, Glyma.13G203900, could be the common major gene of these two QTL, for it was involved in the flavonoid biosynthetic process according to the GO database. Meanwhile, the Glyma.13G202500 and Glyma.13G203800, which could influence the accumulation of anthocyanin and lignin, were also the candidates, for the anthocyanin and lignin had the common precursors of isoflavones and obstructing the anthocyanin branch pathway could promote the accumulation of isoflavones (Yu et al., 2003).

One interesting finding was that a short region on the 7th chromosome contained three relational QTL, which were qAGLME072 (34.56–36.54 cm), qGEEME071 (34.89–36.84 cm), and qGLEME071 (34.96–36.85 cm). qAGLME072 were derived from qAGLm05 (Primomo et al., 2005) and qAGLm12 (Zeng et al., 2009). qGEEME071 was obtained from 5 original QTL including qGEEm02, qGEEm03 (Primomo et al., 2005), qGEEm11 (Zeng et al., 2009), qGEEm52 (Wang et al., 2015), and qGEEm55 (Han et al., 2015). qGLEME071 was analyzed based on 6 original QTL: qGLEm06, qGLEm07 (Primomo et al., 2005), qGLEm11 (Zeng et al., 2009), qGLEm58, qGLEm60 (Wang et al., 2015), and qGLEm61 (Han et al., 2015). These original QTLs were mapped from the 11 environments and two pairs of parental lines: AC756 × RCAT Angora and Zhongdou27 × Jiunong20. This study supported that they were the same QTL, which was similar to qGEEME062 and qAGLME061. Combing with the results of gene calling and functional annotation, this research further inferred that this region may contain gene(s) directly related to the isoflavonoid biosynthesis pathway, for the candidate gene, Glyma.07G112700, located on this region could encode a protein with 4-coumarate-CoA ligase (4CL) activity. The 4CL, a key enzyme in the phenylalanine metabolic pathway (Yu et al., 2003), could directly catalyze the biosynthesis of lignin which was the precursor of aglycone isoflavones. This implied that our assumption might be true. The previous research identified a total of four *4cl* genes in soybean: *Gm4CL1* (on the 17th chromosome), *Gm4CL2* (on the 13th chromosome), *Gm4CL3* (on the 11th chromosome), and *Gm4CL4* (on the 1st chromosome) (Lindermayr et al., 2002). Therefore, Glyma.07G112700 might be a new homologous gene of the *Gm4CL* family. Meanwhile, this research did not exclude the possibility that these QTL could affect the aglycone isoflavones indirectly. Such as Glyma.07G111700, a candidate gene in the interval, may encode a protein with protein kinase activity, which might regulate the activity of several enzymes in the isoflavone biosynthesis pathway like GmMPK1 (Wu et al., 2020).

### Novel Quantitative Trait Locus Identification

Of the 41 QTL mapped in this paper, 38 QTL were novel while three (qPRO0902, qPRO1101, and qOIL1702) had been identified in previous research (Tajuddin et al., 2003; Liang et al., 2010; Lu et al., 2013). This study identified the three reported QTL with smaller interval length (0.00–0.45 cm), while the previous researches had mapped QTL with bigger interval (8.30–20.00 cm) (Hyten et al., 2004; Liang et al., 2010; Lu et al., 2013). Meanwhile, these three reported QTL also indirectly identified that the major genes of these QTL could be effective on multiple conditions, for the experimental conditions of both current and previous studies were different. Consequently, these QTL and its major genes might be greatly useful for soybean genetic improvement. Additionally, all other QTL, except qPRO0201 (12.32 cm), were also identified in the smaller interval (0.32–0.97 cm) compared with the previous researches of (1.35–101.74 cm) (Gutierrez-Gonzalez et al., 2010, 2011; Leite et al., 2016; Zhou et al., 2016; Pei et al., 2018; Lee et al., 2019). The smaller interval of QTL will promote the investigation of major genes, as Liang (2021) demonstrated that the more precise and accurate interval of QTL could make QTL more closely to the quantitative trait gene (QTG) (Liang et al., 2021). Furthermore, though the several QTL mapped in the previous researches contained short interval length (less than 1 cm) as well, the QTL mapped in this paper had greater LODs (average 8.73) compared with the previous researches (2.30–8.85) (Primomo et al., 2005; Pathan et al., 2013; Wang et al., 2015; Asekova et al., 2016; Wu et al., 2020). This indicated that the QTLs of the present study were more credible. The most vital reason for this must be the application of high-density molecular markers and DNA resequencing technology in this study. The QTL identified in this study depended on a high-density genetic map constructed by 1,093,273 SNP makers, while low-density genetic maps and SSR and RFLP markers were utilized in the previous studies (Liang et al., 2010; Lu et al., 2013; Mao et al., 2013). In addition, the PVE of qDA0403, qGL1102, qDA0403, qGLU0403, qAGL0403, qTIF0403, qDA0502, qDAE0502, qGLU0502, and qTIF0502 was high and stable in various experimental locations (Table 5). This indicates that the effect of these QTL is significant and not affected by the environment. It could be further inferred that these QTL might contain several major genes which are environmental-independent and could control these quality traits. Therefore, these QTL and their major genes could be useful for and breeding.

There were two stable QTL, qDAE0403 and qGL1102, which exhibited significant LOD scores in the three environments. This demonstrated that these QTLs were stable in multiple environmental conditions. It could be further inferred that the expression and functions of these QTLs might not be affected by environmental change. These two QTLs have crucial guiding significance in constructing stable molecular modular of great quality traits in breeding soybean cultivars. One of the stable QTL, qGL1102, was partly overlapped with a reported QTL, KGl_2 (associated with GLE derivatives contents, the total content of GEE, GE, acetylgenistin, and malonylgenistin) (Yoshikawa et al., 2010). We argued that qGL1102 and KGl_2 might work together to participate in the pathway or contain common major genes, based on the following reasons: (1) acetylgenistin and malonylgenistin derived from GL, and GLE were the precursor of GL; (2) there were few candidate genes on the non-overlapping region between qGL1102 and KGl_2. This study also supported that the major genes of and KGl_2 were regulatory genes, for many candidate genes obtained from them involved the regulation process, such as RNA splicing. The other stable QTL, qDAE0403, was located in the region of 50734438 bp and 51188701 bp on the 4th chromosome. Five QTL were also mapped in this region, including qDA0403, qGLE0403, qGLU0403, qAGL0403, and qTIF0403. With the combination of the functional annotation, this study supposed that *Glyma.04G244100.1* and (or) *Glyma.04G239000.1* were the common QTG of these six QTL. Because both these genes participated in the glycometabolism and encode enzymes with protein serine/threonine kinase activity (GO:0004674), according to the GO database. Although these enzymes does not participate in the isoflavones biosynthesis pathway directly, they may regulate the activity of sucrose synthase (SUS) by phosphorylating and dephosphorylating the serine (Huber et al., 1996), while the SUS could catalyze the biosynthesis of UDP-Glucose which could react with aglycones to form the glucoside isoflavones (Köster and Barz, 1981). Therefore, the candidate genes of these six QTL, *Glyma.04G244100.1* and (or) *Glyma.04G239000.1*, could influence both the *catabolism of aglycones* and *anabolism of glucosides* by *regulating the biosynthesis of the UDP-Glucose*, and thus, may influence both the individual and total isoflavone contents. In addition, the Glyma.04G242200.1, Glyma.04G239000.1, and (or) Glyma.04G239000.2 could be the major genes as well, for they are involved in the process of response to auxin (GO:0009733) which is a phytohormone influencing the accumulation of isoflavones (Luczkiewicz et al., 2014).

The present study also found several QTLs linked with different traits have existed in the same regions. This demonstrated that the genes located on these QTL could play an important role in the isoflavone biosynthesis pathway, such as encoding key enzymes that participated in the biosynthesis pathway directly, encoding transcription factors controlling a key step of the pathway indirectly, or regulating an important reaction in epigenetic levels mediately.

One unanticipated finding was that both qAGL0501 and qOIL0501 was located in the same region, and both the additive effect of qAGL0501 (6.00) and qOIL0501 (0.14) was positive in Harbin, which confirmed the results of the previous research that positive correlations between oil and aglycone might be existed genetically (Han et al., 2015). These QTL may contain *several genes, respectively, related to isoflavones and oil contents*, or contain several genes *involved in both the isoflavones and oil biosynthetic pathways*. Additionally, this study also found that the qAGL0501 exhibited significant LOD scores in the Hailun location, but the additive effect was –183.95 in Hailun. Based on the striking contrast between the additive effect of qAGL0501 in Hailun and Harbin, it is hypothesized that qAGL0501 might be *regulated by temperature* or involved in the *response to low-temperature stress*. The gene annotation may verify these inferences. According to the KEGG database, the qAGL0501 may participate in the plant hormone signal transduction pathway (pathway ID: ko04075) and the candidate gene (Glyma.05G241600) were involved in the pathway. Further combining with the Nr database and GO database, these genes may synthesize *histidine kinase 4-like protein* and involve 59 biological processes including the *regulation of anthocyanin metabolic process* (GO:0031537), *cellular response to cold* (GO:0070417), and *fatty acid beta-oxidation* (GO:0006635). It is well-known that the anthocyanin metabolic process is a pathway closely related to genistein biosynthesis (Yu et al., 2003), and the fatty acid beta-oxidation pathway is the key process of oil accumulation. The positive additive effect of qAGL0501 in Harbin (6.00) but the negative effect in Hailun (–183.95) might be due to the differences in cold stress response. Therefore, Glyma.05G241600 is the common major gene of qAGL0501 and qOIL0501 and is involved in three biological processes including *isoflavone biosynthesis*, *oil accumulation*, and *response to low-temperature stress*.

The QTL, qPRO0902, involved any pathway according to the KEGG database. We supposed that qPRO0902 was a false-positive result based on the following reasons: (1) qPRO0902 was not involved in any pathway according to the annotation with the KEGG database in the present study; (2) the LOD of qPRO0902 was three which did not indicate an extremely significant position; (3) the three candidate genes (Glyma.09G054500, Glyma.09G054600, and Glyma.09G054700) of qPRO0902 could not encode characterized protein, according to the BlastX. Nevertheless, the genes of qPRO0902 may contain new functions, for the annotation based on the Swissport database indicated that Glyma.09G054700 had homologous genes in mice.

### Summary and Further Research Avenue

Isoflavone, soy protein, and soybean oil are momentous quality traits in soybean breeding, for they play a crucial role in human health. In this study, the high-density genetic map constructed by whole-genome resequencing was used, and the QTL with short intervals were mapped. The expression analysis based on the public transcriptome data was adopted, and several major genes has been predicted. For the first time, the meta-analysis method was used to investigate the genetic background of isoflavones. A total of 41 QTL (containing 660 genes) associated with 12 kinds of quality traits were obtained on the basis of linkage analysis between phenotypes and the high-density genetic map of ZH RIL, and a total of 212 meta-QTL were mapped based on the public genetic map and meta-analysis.

This finding could promote the understandings of the biosynthesis and regulation of isoflavones, protein, and oil more clearly at the molecular level, and facilitate the molecular breeding for great quality traits in soybean. Meanwhile, fine-mapping qPRO0201 using secondary mapping populations from ZH RIL, and candidate genes identified using the RNA-seq with different expression analysis (DE analysis) and weighted gene co-expression network analysis (WGCNA) could be required for future research.

## Data Availability Statement

The data presented in the study are deposited in the NCBI repository, accession numbers: PRJNA778303 and PRJNA778408.

## Author Contributions

XP, FW, and QZ designed the research. HC, CL, XW, and YL conducted the experiments and analyzed the data. HC and XP conducted the field trial. HC and QZ wrote the manuscript. All authors read and approved the manuscript.

## Conflict of Interest

The authors declare that the research was conducted in the absence of any commercial or financial relationships that could be construed as a potential conflict of interest.

## Publisher’s Note

All claims expressed in this article are solely those of the authors and do not necessarily represent those of their affiliated organizations, or those of the publisher, the editors and the reviewers. Any product that may be evaluated in this article, or claim that may be made by its manufacturer, is not guaranteed or endorsed by the publisher.

## Footnotes

1
https://www.soybase.org/

2
https://www.ncbi.nlm.nih.gov/genome/?term=soybean

3
https://gatk.broadinstitute.org/hc/en-us#variant-discovery-ovw

4
http://samtools.sourceforge.net/

5
https://www.soybase.org/dlpages/gmapdownload.php

6
https://urgi.versailles.inra.fr/content/download/2263/20262/file/UserGuideBioMercatorV4.pdf

7
https://jgi.doe.gov/data-and-tools/phytozome/

8
https://www.kegg.jp/kegg/

9
https://blast.ncbi.nlm.nih.gov/

10
http://www.ncbi.nlm.nih.gov/COG/

11
http://www.gpmaw.com/html/swiss-prot.html
