# Supplementary material for: Novel QTL and Meta-QTL Mapping for Major Quality Traits in Soybean
Source: Front Plant Sci. 2021 Dec 8;12:774270. doi: 10.3389/fpls.2021.774270 (PMC8692671; doi:10.3389/fpls.2021.774270)
Supplement: Supplementary file 1 [file Data_Sheet_1.PDF]

Fig. S1 The frequency distribution histogram of 12 quality traits contents in the three experimental locations

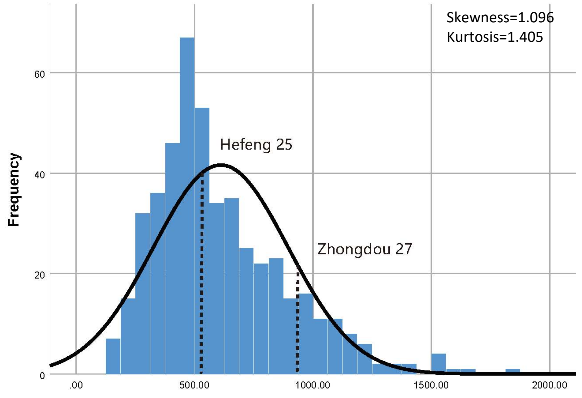

(1a) Daidzin-Harbin

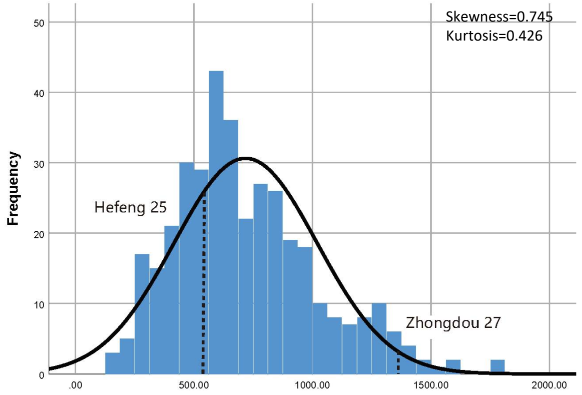

(1b) Daidzin-Hailun

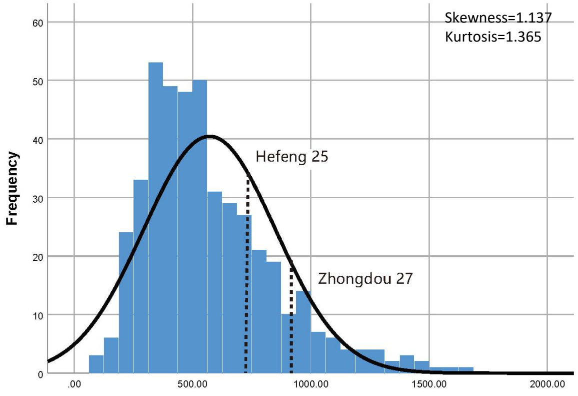

(1c) Daidzin-Mudanjiang

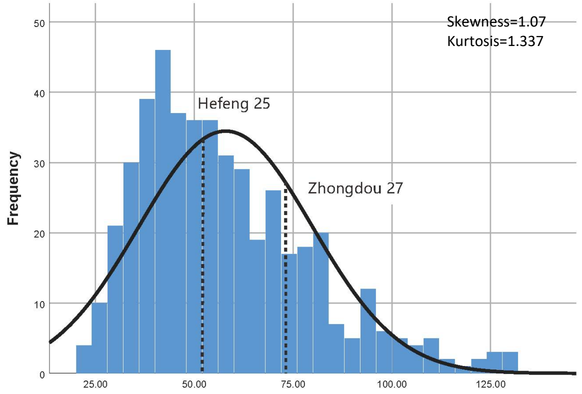

(2a) Glycitin-Harbin

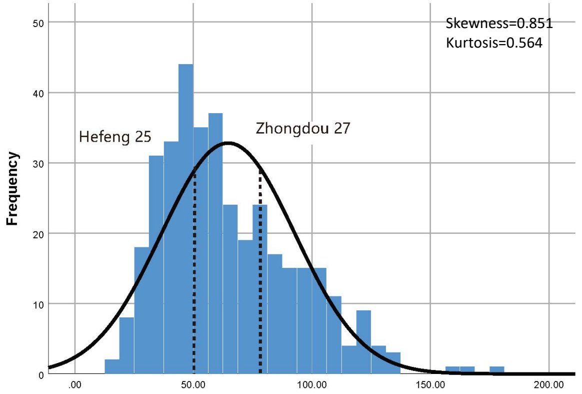

(2b) Glycitin-Hailun

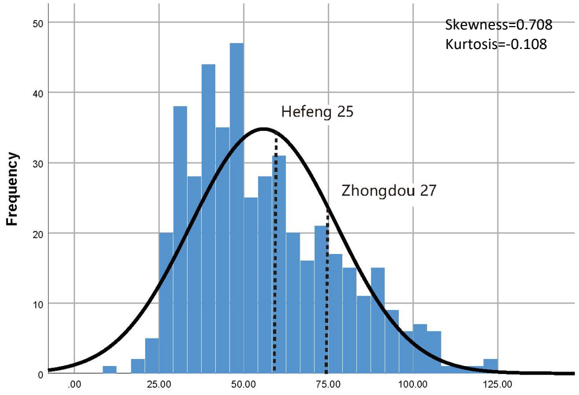

(2c) Glycitin-Mudanjiang

11111

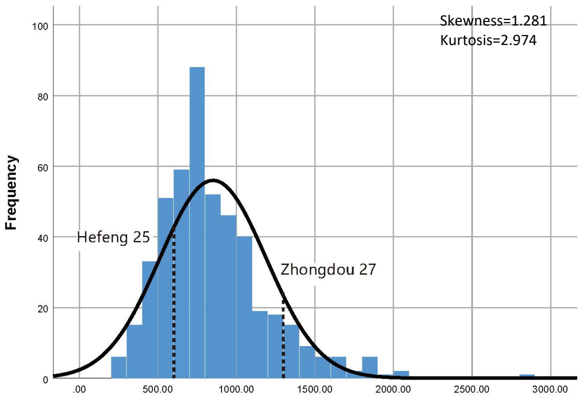

(3a) Genistin-Harbin

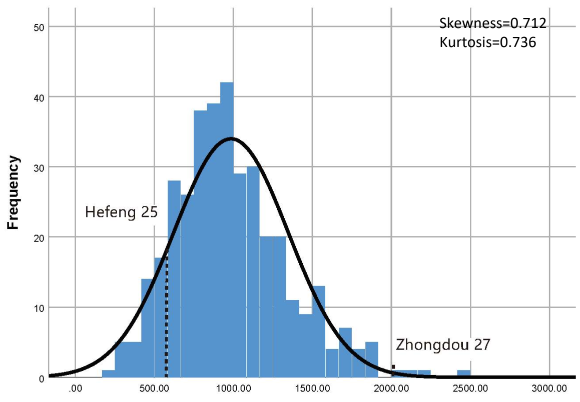

(3b) Genistin-Hailun

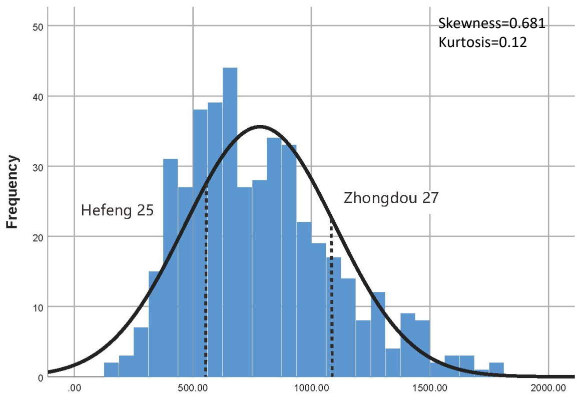

(3c) Genistin-Mudanjiang

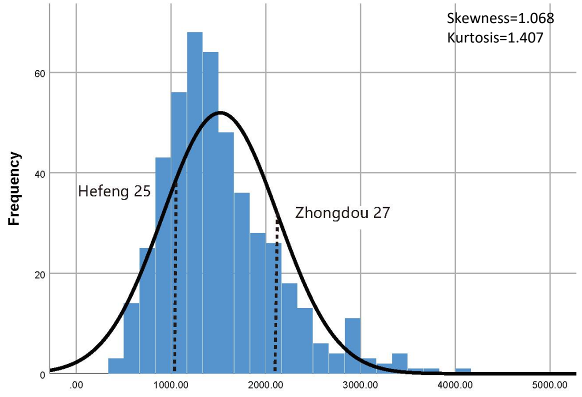

(4a) Glucoside-Harbin

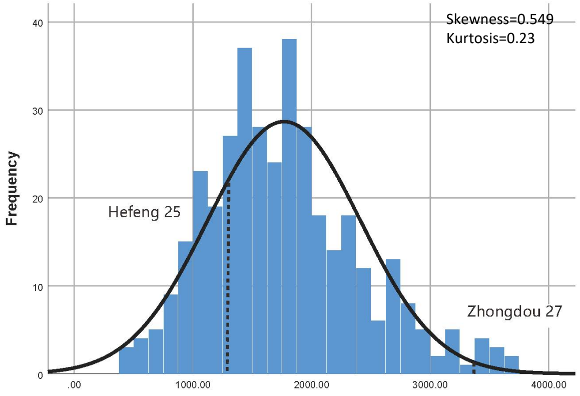

(4b) Glucoside-Hailun

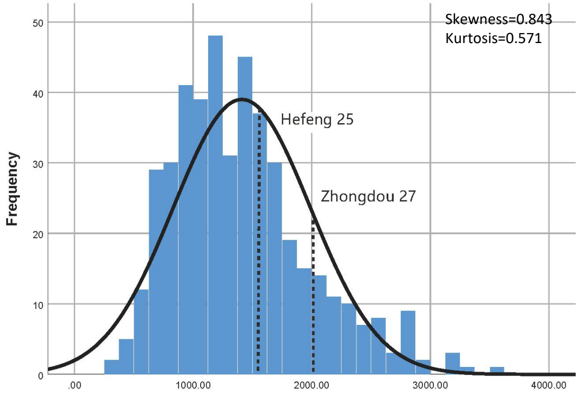

(4c) Glucoside-Mudanjiang

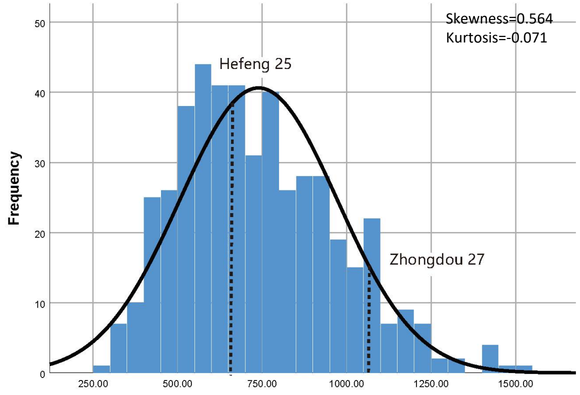

(5a) Daidzein-Harbin

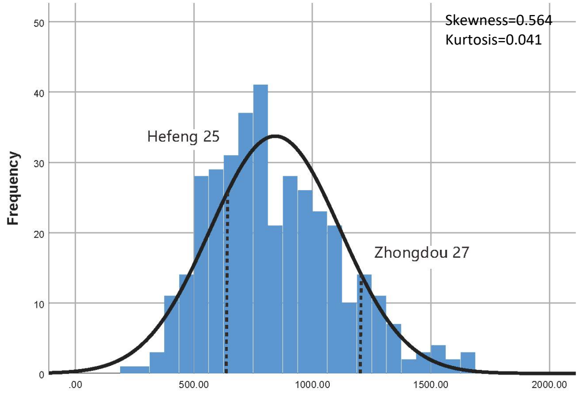

(5b) Daidzein-Hailun

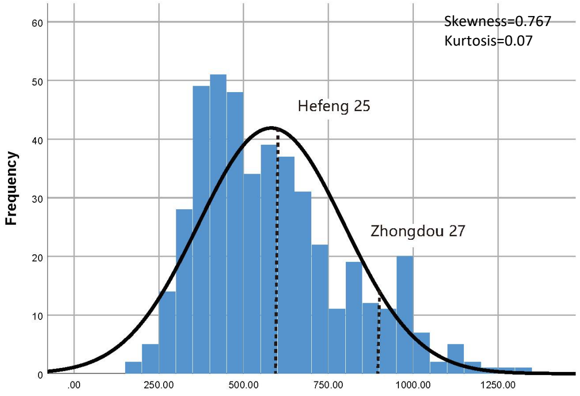

(5c) Daidzein-Mudanjiang

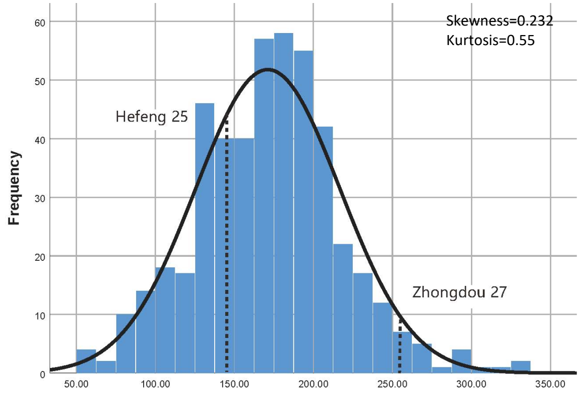

(6a) Glycitein-Harbin

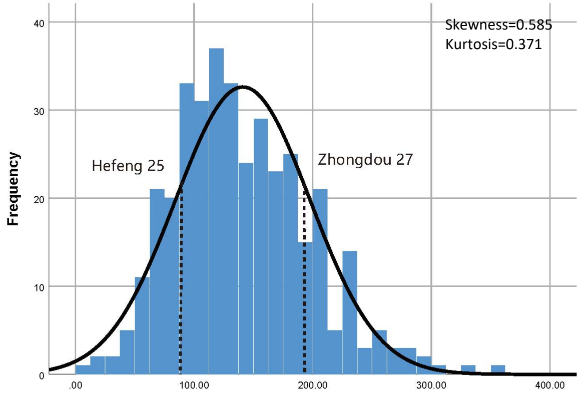

(6b) Glycitein-Hailun

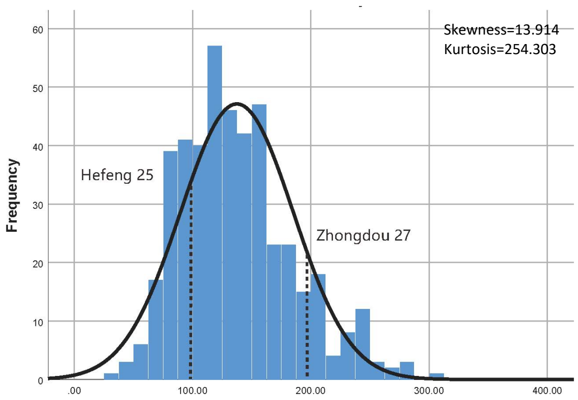

(6c) Glycitein-Mudanjiang

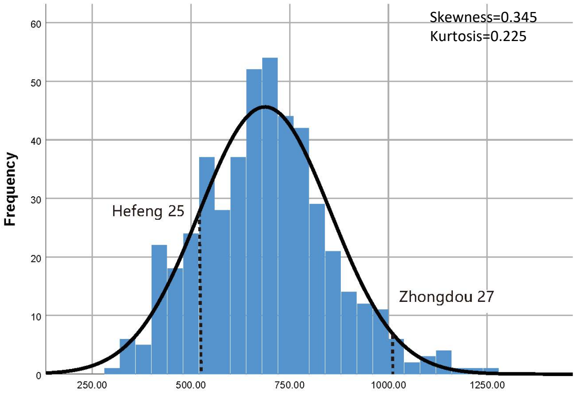

(7a) Genistein-Harbin

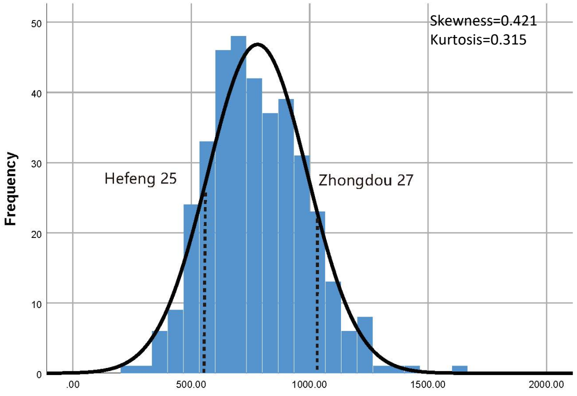

(7b) Genistein-Hailun

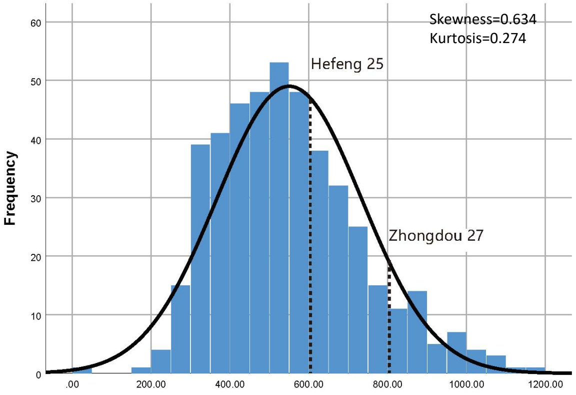

(7c) Genistein-Mudanjiang

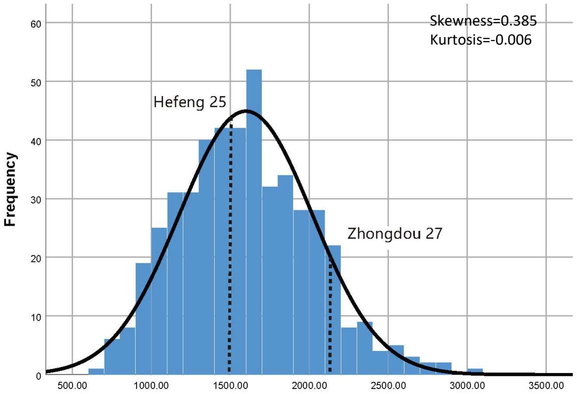

(8a) Aglycone-Harbin

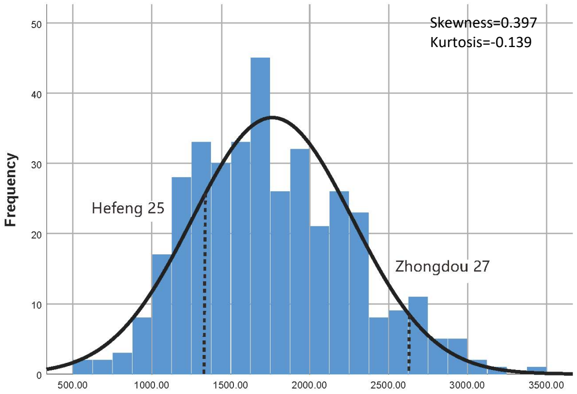

(8b) Aglycone-Hailun

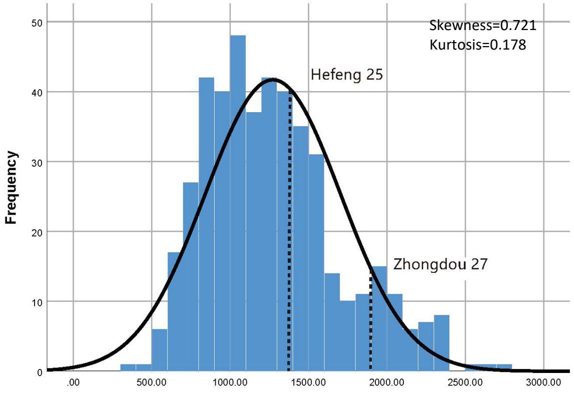

(8c) Aglycone-Mudanjiang

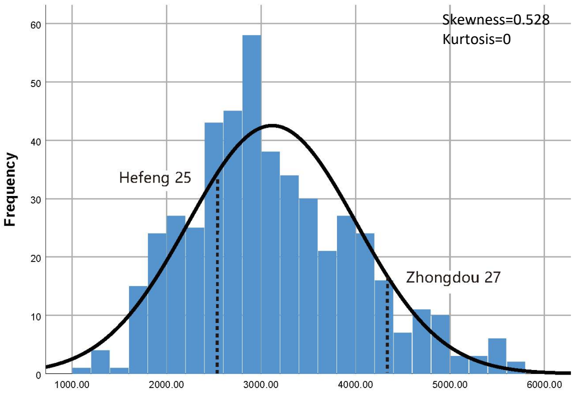

(9a) TIF-Harbin

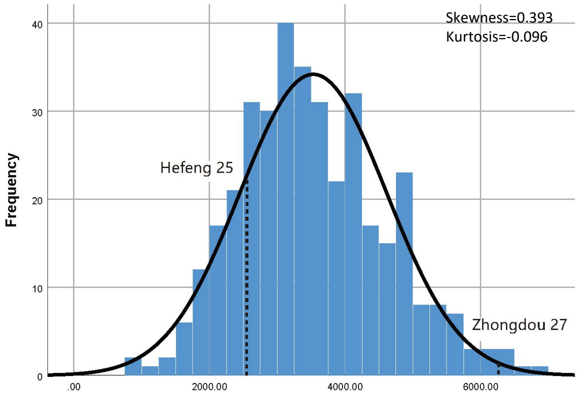

(9b) TIF-Hailun

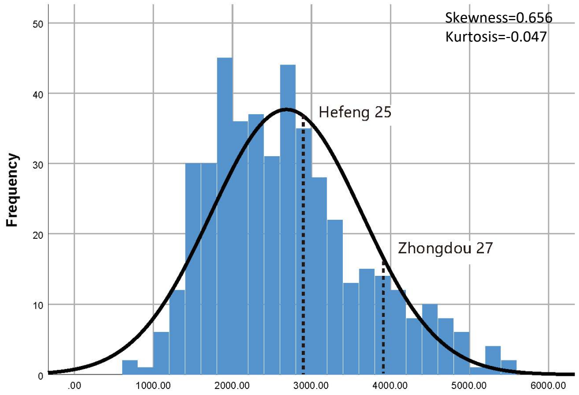

(9c) TIF-Mudanjiang

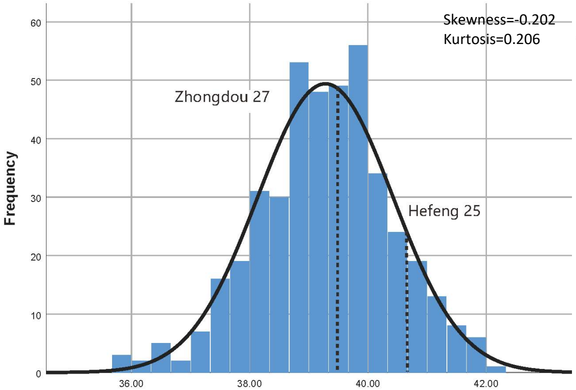

(10a) Protein-Harbin

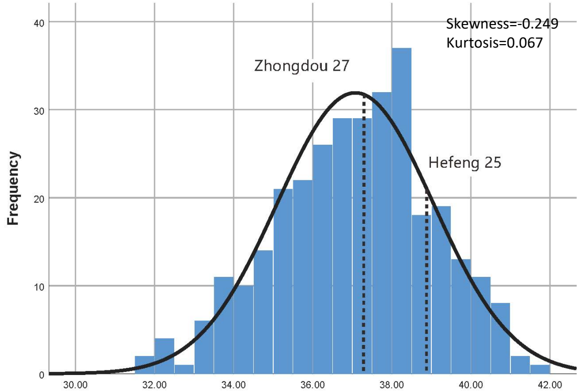

(10b) Protein-Hailun

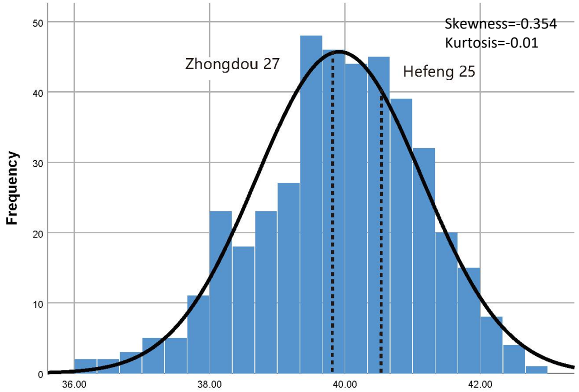

(10c) Protein-Mudanjiang

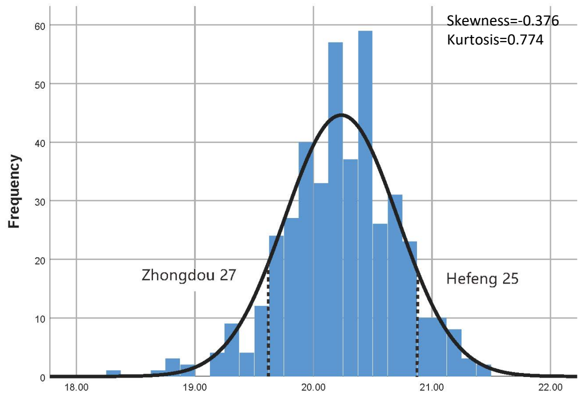

(11a) Oil-Harbin

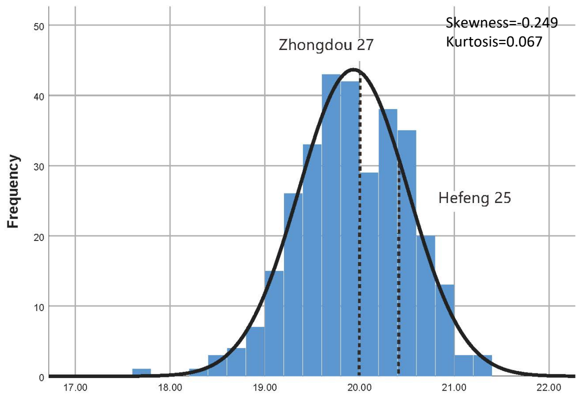

(11b) Oil-Hailun

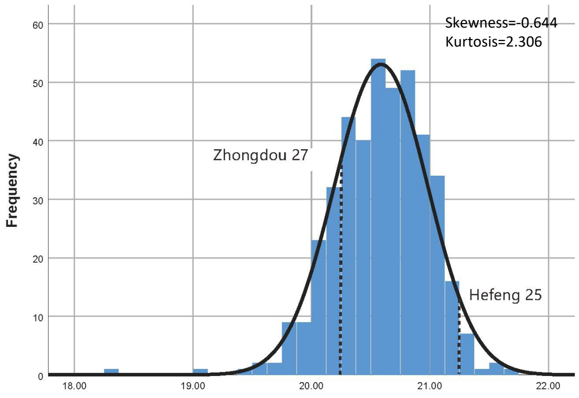

(11c) Oil-Mudanjiang

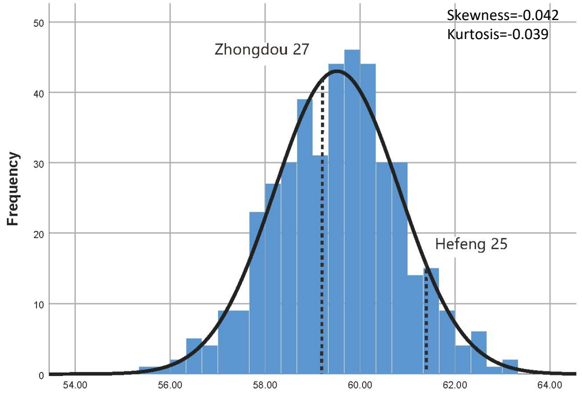

(12a) P+O-Harbin

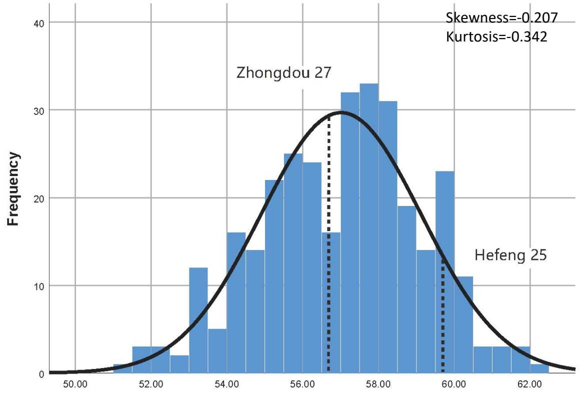

(12b) P+O-Hailun

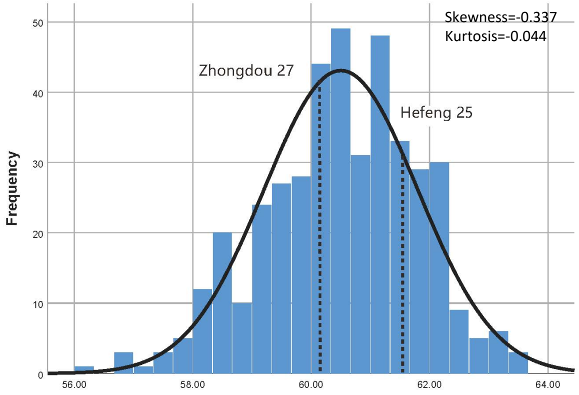

(12c) P+O-Mudanjiang
